# *In situ* synthesis, crystal structures, topology and photoluminescent properties of poly[di-μ-aqua-di­aqua­[μ_3_-4-(1*H*-tetra­zol-1-id-5-yl)benzoato-κ^4^ *O*:*O*,*O*′:*O*′′]barium(II)] and poly[μ-aqua-di­aqua­[μ_3_-4-(1*H*-tetra­zol-1-id-5-yl)benzoato-κ^4^ *O*:*O*,*O*′:*O*′]strontium(II)]

**DOI:** 10.1107/S2056989020006386

**Published:** 2020-05-19

**Authors:** Mohamed Abdellatif Bensegueni, Aouatef Cherouana, Hocine Merazig

**Affiliations:** a Environmental, Molecular and Structural Chemistry Research Unit, University of Constantine-1, 25000, Constantine, Algeria

**Keywords:** alkaline earth complexes, tetra­zol-carboxyl­ate coordination compounds, *in situ* synthesis, photoluminescence, TGA, FT–IR, topology, crystal structure

## Abstract

Two alkaline-earth coordination compounds, [Ba(C_8_H_4_N_4_O_2_)(H_2_O)_4_]_*n*_, (I), and [Sr(C_8_H_4_N_4_O_2_)(H_2_O)_3_]_*n*_, (II), from the one-pot hydrolysis transformation of benzoyl chloride and the *in situ* self-assembled [2 + 3] cyclo­addition of nitrile are presented. These coordination compounds are prepared by reacting 4-cyano­benzoyl chloride with divalent alkaline-earth salts (BaCl_2_ and SrCl_2_) in aqueous solution under hydro­thermal conditions. The mononuclear coordination compounds (I) and (II) show the same mode of coordination of the organic ligands. The cohesion of the crystalline structures is provided by hydrogen bonds and π-stacking inter­actions, thus forming three-dimensional supra­molecular networks. The two compounds have a three-dimensional (3,6)-connected topology, and the structural differences between them is in the number of water mol­ecules around the alkaline earth metals. Having the same emission frequencies, the compounds exhibit photoluminescence properties with a downward absorption value from (I) to (II).

## Abstract 2

The crystal structures of two alkaline-earth-based coordination compounds containing bifunctional tetra­zole­carboxyl­ate ligands reveal a one-dimensional chain structure with the carboxyl­ate-tetra­zole anionic ligand. The structures of both compounds are dominated by hydrogen bonds involving water coordination ligands, with an underlying 3.6 − *c* network.

## Chemical context   

In recent years, studies on a wide variety of tetra­zolyl-5-substituted coordination compounds have proliferated (Klapötke & Stierstorfer, 2009 ▸; Fischer *et al.*, 2011 ▸). The extension from the synthetic approach developed by Demko and Sharpless (2001 ▸) to that of Zhao and colleagues (Zhao *et al.*, 2008 ▸) is the main reason for this new inter­est. Chemists have focused on transition-metal compounds, while studies with alkaline-earth metal–tetra­zol coordination compounds remain scarce. This led us to further explore this type of compound, and to study their topological and physical properties.

The choice of ligand is essential in the design of new coordination compounds. In our study we selected a (tetra­zol-carboxyl­ate) bifunctional ligand, which is able to adopt several coordination modes, resulting in a variety of crystal structures (Ouellette *et al.*, 2012 ▸; Sun *et al.*, 2013 ▸; Wei *et al.*, 2012 ▸).

The complexation and formation of both the tetra­zole and carboxyl­ate groups occurred *in situ* under hydro­thermal conditions from a 4-cyano-benzoyl chloride and the alkaline earth salts BaCl_2_·2H_2_O and SrCl_2_·6H_2_O, giving the title compounds poly[di-μ-aqua-di­aqua­[μ_3_-5-(4-carboxyl­ato­phen­yl)-1*H*-1,2,3,4-tetra­zol-1-ido-κ^4^ *O*:*O*,*O*′:*O*′′]barium(II)] (I) and poly[μ-aqua-di­aqua­[μ_3_-4-(1*H*-tetra­zol-1-id-5-yl)benzoato-κ^4^ *O*:*O*,*O*′:*O*′]strontium(II)] (II). The two compounds form one-dimensional crystalline chains, in which the coordination is ensured by chelating carboxyl­ate groups. The two compounds were characterized by FT–IR, TGA and single-crystal X-ray diffraction analysis. A topological study was performed and the photoluminescent properties were also studied.
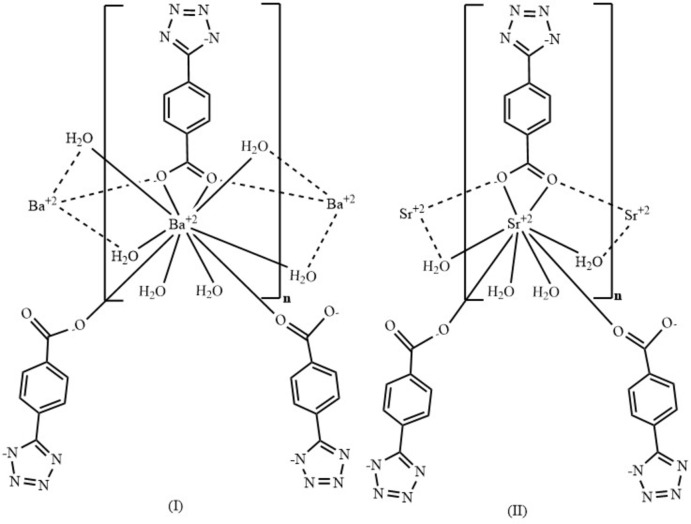


## Structural commentary   

**Figure 1:**
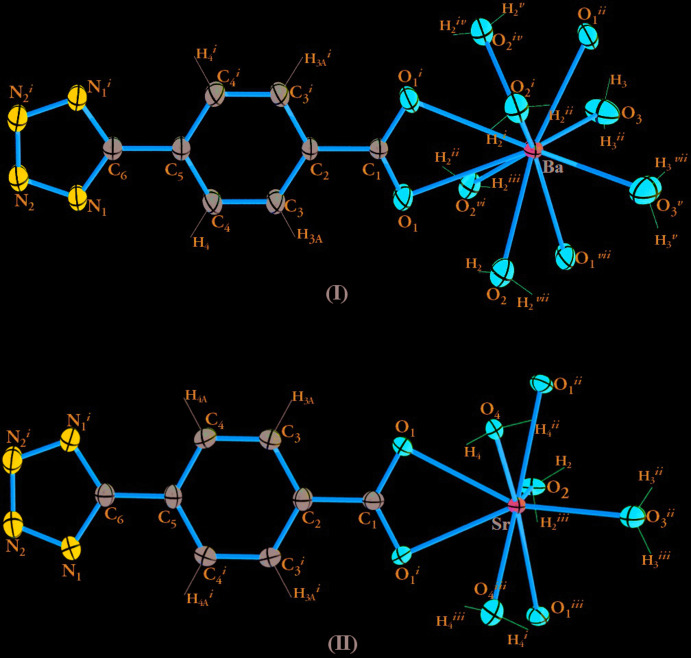
The coordination environment of the *Ae* ^2+^ ion in compounds (I) and (II), showing the atom-numbering scheme. Displacement ellipsoids are drawn at the 50% probability level. [Symmetry codes for (I): (i) 2 − *x*, 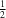 − *y*, *z*; (ii) 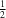 + *x*, 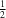 − *y*, 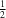 − *z*; (iii) *x* − 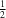, 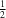 − *y*, 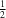 − *z*; (iv) 2 − *x*, *y*, *z*; (v) 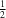 + *x*, *y*, 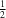 − *z*; (vi) *x* − 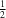, −*y*, *z*; (vii) −*x* − 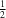, *y*, 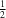 − *z*; and for (II): (i) 2 − *x*, *y*, *z*; (ii) 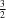 − *x*, *y*, 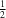 − *z*; (iii) *x* + 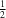, *y*, 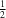 − *z*.]

Compound (I) crystallizes in the ortho­rhom­bic space group *Imma* while compound (II) crystallizes in *Pmna*. In these two coordination compounds, the asymmetric unit comprises half of a crystallographically independent alkaline-earth metal ion, half of a deprotonated 4-(tetrrazol-5-yl)benzoate anion (ttzbenz), and two halves of water mol­ecules in compound (I) and three halves of water mol­ecules in compound (II) (Fig. 1 ▸). The bond distances and angles of the ligands are comparable to those found in the literature for similar systems (Zheng *et al.*, 2009 ▸; Jiang *et al.*, 2007 ▸; Yu *et al.*, 2009 ▸).

**Figure 2:**
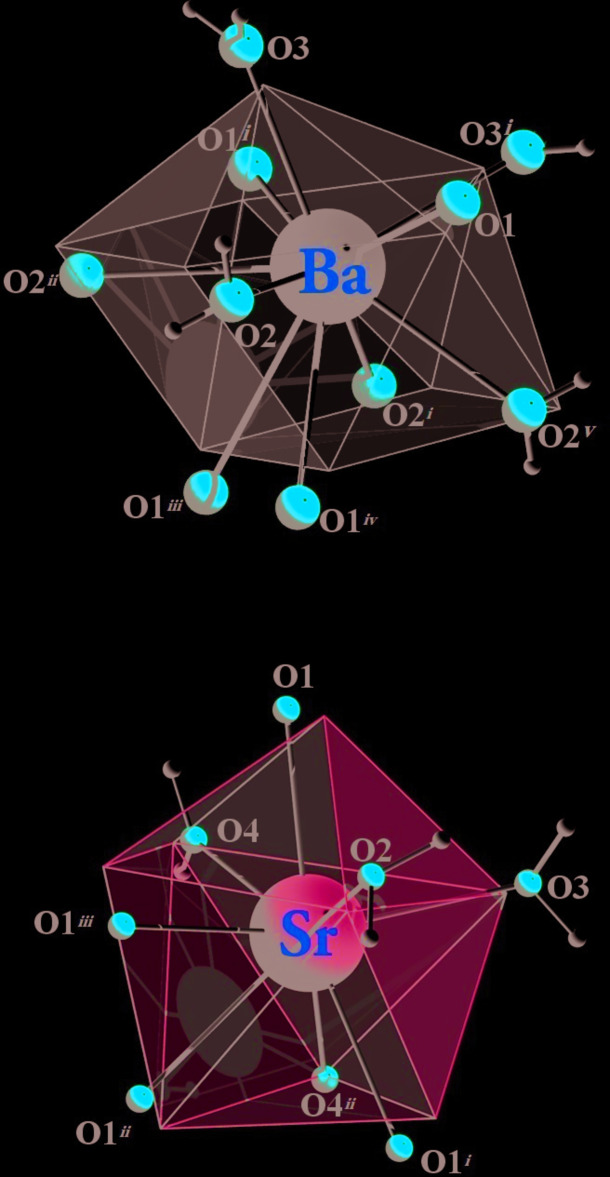
Coordinating polyhedra of compounds (I) and (II), the colored polyhedra with open front faces represent the ideal polyhedral shape as calculated by *SHAPE 2.1* [Symmetry codes for (I): (i) 1 − *x*, 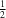 − *y*, *z*; (ii) 1 − *x*, *y*, *z*; (iii) −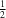 + *x*, 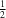 − *y*, 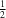 − *z*; (iv) 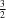 − *x*, *y*, 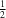 − *z*; (v) *x*, 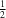 − *y*, *z*; and for (II): (i) 2 − *x*, *y*, *z*; (ii) 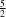 − *x*, *y*, 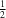 − *z*; (iii) −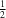 + *x*, *y*, 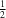 − *z*.]

The crystal structures of compounds (I) and (II) show similar topologies, the main difference being the coordination polyhedron around the metal center. In compound (I), a slightly distorted BaO_10_ sphenocorona coordination geometry (Casanova *et al.*, 2005 ▸) is observed (Fig. 2 ▸). The geometry deviates by 4.424 compared to the theoretical model as proposed by *SHAPE 2.1* software (Casanova *et al.*, 2005 ▸; see Table S1 in the supporting information). In (I), the barium cation is deca­coordinated by four oxygen atoms from three ttzbenz ligands, two independent oxygen atoms from two terminal water mol­ecules (O2 and O3) and four additional oxygens from bridging water mol­ecules. In compound (II), the Sr^2+^ ion is eightfold coordinated, being surrounded by four bridging water mol­ecules and by four oxygen atoms from three symmetry-related ttzbenz ligands (Fig. 2 ▸), thus generating a triangular dodeca­hedral SrO_8_ coordination geometry; this geometry deviates by 3.426 compared to the theoretical model proposed by *SHAPE 2.1* software (Casanova *et al.*, 2005 ▸; see Table S1 in the supporting information).

The bond angles (Tables 1 ▸ and 2 ▸) around the *Ae* ^2+^ ion (*Ae* ^2+^ = Ba^2+^ and Sr^2+^) range between 42.49 (6) and 142.50 (2)° in compound (I), and between 48.93 (6) and 148.91 (4)° in compound (II). The Ba—O bond lengths are 2.821 (2) and 2.875 (1) Å for the coordinated water mol­ecule, and 2.660 (2) and 3.016 (2) Å for the ttzbenz oxygen atom (Table 2 ▸), and these distances are slightly longer than that in an analogous compound (Fu *et al.*, 2010 ▸). The Sr—O bond lengths are 2.501 (2) and 2.660 (1) Å for the ttzbenz oxygen atom, and 2.549 (2) and 2.676 (2) Å for the coordinated water mol­ecule (Table 2 ▸). The Ba—O bonds are longer than Sr—O bonds; this is due not only to the nature of the metal, but also, in part, to the measurement temperature [room temperature for compound (I), but 150K for compound (II). These bond-length values are close to those observed in similar compounds based on *Ae* ^2+^ one-dimensional coordination polymers: Ba—O = 2.647–3.179 Å, Sr—O = 2.486–2.843 Å in [C_24_H_28_N_2_O_13_Cl_2_CuSr]_*n*_ and [C_24_H_28_N_2_O_13_Cl_2_CuBa]_*n*_ (Hari, *et al.*, 2017 ▸), and in the compounds [C_8_H_16_N_16_O_19_Sr_4_]_*n*_ and [C_8_H_20_N_16_O_18_Sr_4_]_*n*_ where the Sr—O distances range from 2.570–2.700 Å and 2.541–2.633 Å, respectively. In the two-dimensional coordination compound [C_2_H_6_BaN_4_O_5_]_*n*_, the Ba—O distances are 2.790 and 2.902 Å (Hartdegen *et al.*, 2009 ▸), while in the three-dimensional polymers [Ba_2_ *M*(HCOO)_6_(H_2_O)_4_]_*n*_, Ba—O = 2.801 (2)–3.6143 (2) Å for *M* = Ni, Ba—O = 2.797 (2)–2.999 (2) Å for *M* = Zn, and Ba—O = 2.801 (2)–3.004 (2) Å for *M =* Co (Baggio *et al.*, 2004 ▸), and in the strontium complex C_6_H_12_SrN_6_O_10_, Sr—O = 2.506–2.724 Å (Divya *et al.*, 2017 ▸).

**Figure 3:**
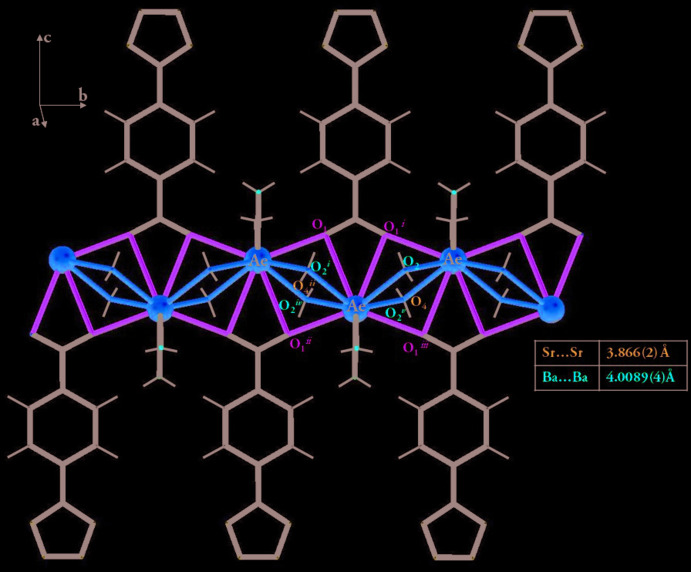
Coordinating polymers along the *b* axis.

The ttzbenz ligand can adopt several coordination modes by involving the tetra­zole ring (Yao *et al.*, 2013 ▸), or the carboxyl­ate group as in our case, where the two compounds use the ttzbenz anion to coordinate two adjacent *Ae* ^2+^ cations in a bidentate chelate manner, thus forming a polyatomic bridge and binding neighboring *Ae* ^2+^ ions in a zigzag manner, resulting in the formation of binuclear units [*Ae*–O1–*Ae*–O1] with a Ba⋯Ba distance of 4.0089 (4) Å for compound (I) and an Sr⋯Sr distance of 3.866 (2) Å for compound (II) (Fig. 3 ▸).

## Supra­molecular features   

**Figure 4:**
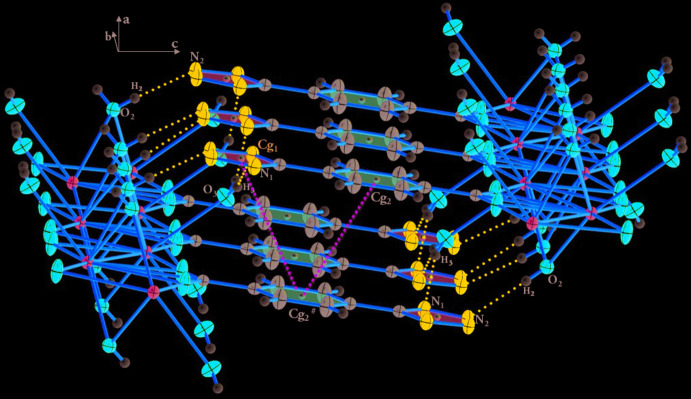
Hydrogen bonds (blue dashed lines) and π-stacking inter­actions (green dashed lines) in the crystal packing of compounds (I) and (II).

In compound (I), hydrogen bonds between two coordinated water mol­ecules and two nitro­gen atoms of the tetra­zole ring of the ttzbenz ligand are observed (Table 3 ▸), ensuring cohesion between the tetra­zole rings and the inorganic [Ba_2_O_2_]_*n*_ chains. In addition to hydrogen bonds, π-stacking inter­actions between phenyl rings are observed (Fig. 4 ▸) with a centroid–centroid distance of 4.035 (1) Å, which enhance the cohesion of the crystal structure.

In compound (II), as well as the strong O—H⋯N hydrogen bonds (Table 4 ▸), weak intra­molecular π-stacking inter­actions are observed, reinforcing the cohesion in the crystal structure between the tetra­zole rings (centroid *Cg*1) and the phenyl rings (centroid *Cg2*) with centroid–centroid distances *Cg*1⋯*Cg*2 = 3.622 (3) Å and *Cg*2⋯*Cg*2 = 3.897 (3) Å (Fig. 4 ▸).

## Topological study   

**Figure 5:**
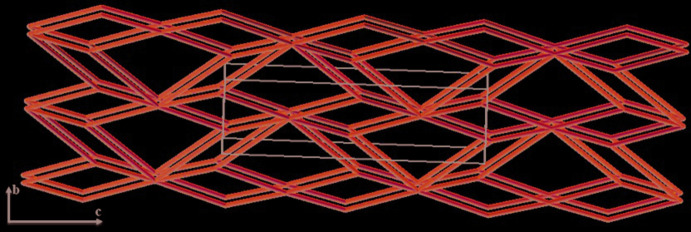
Simplification of the coordination framework in the two compounds using standard representation for valence-bonded CPs.

To simplify the crystalline structure of the title compounds, we used the standard representation of valence-bound CPs (CP = coordination polymer) to obtain the underlying network. In such models, only metal centers and the centroids of organic ligands are considered as structural units (Alexandrov *et al.*, 2011 ▸). The simplification of the crystal structure of the two compounds by this procedure and the topological classification of the two studied compounds led to the same topological network, identified as a 3.6-*c* net with stoichiometry (3-*C*)_2_(6-*C*), which can be represented by the point symbol {4^3^}_2_{4^6^.6^6^.8^3^}. Thus the two structures consist of planar layers running parallel to (100) (Fig. 5 ▸).

## Database survey   

A search for 4-(tetra­zol-5-yl) benzoate in the Cambridge Structural Database (CSD Version 5.40; Groom *et al.*, 2016 ▸) gave 81 hits for the ligand, alone or with co-ligands. The ttzbenz ligand has proved to be an excellent component for the assembly of new coordination complexes and polymers, whether through a bridging and/or chelating coordination mode, mono or polydentate, and as an acceptor of hydrogen bonds through the two carboxyl­ate and tetra­zolate groups. This has led to structural diversity with inter­esting physicochemical properties, as seen in the structures with metal ions: copper (Ouellette *et al.*, 2009 ▸), cobalt (Ouellette *et al.*, 2012 ▸), zinc (Wei *et al.*, 2012 ▸; Jiang *et al.*, 2007 ▸; Zheng *et al.*, 2009 ▸), lead (Sun *et al.*, 2013 ▸), manganese and cadmium (Cheng *et al.*, 2016 ▸; Yu *et al.*, 2009 ▸), europium, terbium (Wang *et al.*, 2011 ▸). Finally, with bi­pyridine co-ligands (Yang *et al.*, 2017 ▸; Gao *et al.*, 2016 ▸), (terpyridin­yl)benzoate (Zhang *et al.*, 2016 ▸), phenanthroline (Werrett *et al.*, 2015 ▸), 3,5-dimethyl-1,2,4-triazolato (Sheng *et al.*, 2016 ▸), and *N*,*N*-di­methyl­acetamide (Wang *et al.*, 2015 ▸).

## Synthesis and crystallization   

Colorless crystals suitable for X-ray diffraction were obtained by hydro­thermal synthesis in an aqueous solution according to a literature procedure (Demko & Sharpless, 2001 ▸; Zhao *et al.*, 2008 ▸), where an aqueous solution (10 ml) of sodium azide (0.065 g, 1 mmol) and 4-cyano­benzoyl chloride (0.165 g, 1 mmol) was added dropwise to an aqueous solution (5 ml) of BaCl_2_·2H_2_O (0.244 g, 1mmol) for (I) and SrCl_2_·6H_2_O (0.266g, 1 mmol) for (II) under constant stirring for a few minutes. The resulting solution was sealed in a 25ml teflon-lined stainless steel autoclave and heated at 453 K for 3 d.

The FT–IR spectra for compounds (I) and (II) were recorded in the frequency range 4000–400 cm^−1^ on a Perkin Elmer FT–IR spectrophotometer Spectrum 1000. The ν, γ and δ modes are: stretching, out-of-plane bending, and in-plane bending, respectively. The absence of bands in the two regions: 2200–2280 cm^−1^ and 2100–2270 cm^−1^ corresponding to the functions –CN and N_3_^−^, respectively, confirms that the [2 + 3] cyclo­addition reaction between the cyano group and the azide anions occurred and the tetra­zolate ligand was formed (Hammerl *et al.*, 2002 ▸, 2003 ▸; Damavarapu *et al.*, 2010 ▸; Zhang *et al.*, 2013 ▸)

FT–IR of (I) (ATR, cm^−1^): 3300 ν(O—H)_water_, 3100 ν(C—H)_Ph_, 1435 ν_sym_ (C—C), 1523 ν(N—N)_ring_, 1603 ν(C—N)_ring_, 628–1050 γ,δ (tetra­zole).

FT–IR of (II) (ATR, cm^−1^): 3600 ν(O—H)_water_, 3200 ν(C—H)_Ph_, 1408 ν_sym_ (C—C), 1530 ν(N—N)_ring_, 1585 ν(C—N)_ring_, 654–1009 γ, δ (tetra­zole) (see Fig. S1 in the supporting information).

**Figure 6:**
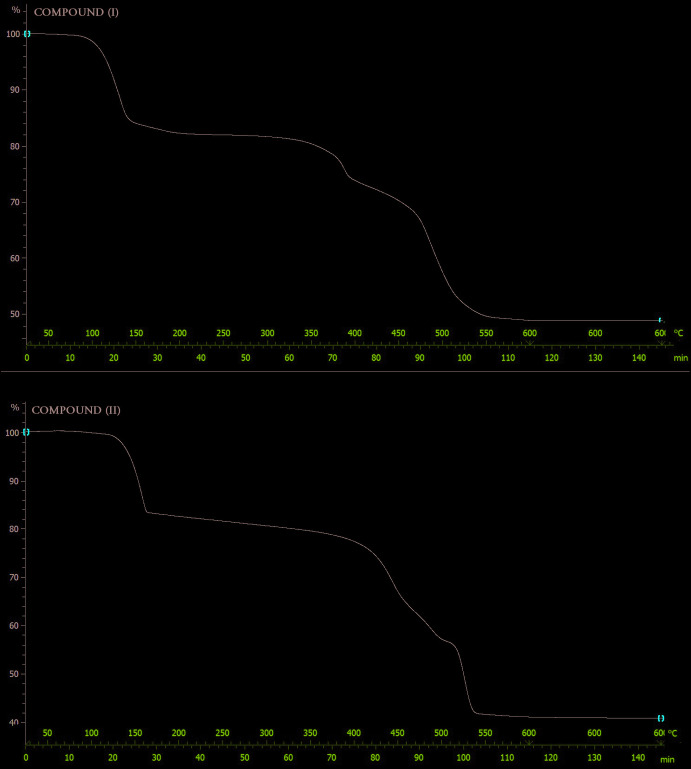
Thermogravimetric analysis of compounds (I) and (II).

The thermogravimetric analysis (TGA) was performed in the range 25–600°C under air atmosphere at a flow rate of 5°C/min (Fig. 6 ▸). The pyrolytic processes for compound (I) occurs in two main steps. The first step corresponds to the release of four water mol­ecules (2 bridging water mol­ecules and 2 monodentate) (scheme1) between 90°C and 200°C, which corresponds to approximately 18% of the weight of (I). Subsequently, the ligands undergo pyrolysis to result in decomposition (32% by weight) in the range of 200 to 600°C. In compound (II), the pyrolytic processes also go through two stages. The first step corresponds to the release of three water mol­ecules (1 bridging water mol­ecule and 2 monodentate) (scheme1) between 100°C and 160°C, which corresponds to approximately 16% of the weight of (II). The second step corresponding to a weight loss of 44% of (II) is attributed to the decomposition of the ligand 160 and 600°C.

## Thermogravimetric analysis   

The thermogravimetric analysis (TGA) was performed in the range 25–600°C under an air atmosphere at a flow rate of 5°C min^−1^ (Fig. 6 ▸). The pyrolytic processes for compound (I) occur in two main steps. The first step corresponds to the release of four water mol­ecules (two bridging water mol­ecules and two monodentate) between 90°C and 200°C, which corresponds to approximately 18% of the weight of (I). Subsequently, the ligands undergo pyrolysis to result in decomposition (32% by weight) in the range 200–600°C. In compound (II), the pyrolytic processes also go through two stages. The first step corresponds to the release of three water mol­ecules (one bridging water mol­ecule and two monodentate) between 100°C and 160°C, which corresponds to approximately 16% of the weight of (II). The second step corresponding to a weight loss of 44% of (II) is attributed to the decomposition of the ligand between 160 and 600°C.

## Fluorescence properties   

The fluorescence properties of compounds (I) and (II) were determined from the emission spectra at the same excitation wavelength (*eX* = 322 nm) on an Agilent Cary Eclipse Fluorescence Spectrophotometer at room temperature. Excitation of the two compounds after dissolution in DMSO leads to similar fluorescence emission spectra. The emission maximum of (I) is observed to shift from 368 to 377 nm and from 371 to 378 nm for II (see Fig. S2 in the supporting information), probably corresponding to π* → π or π*→n electronic transition of the aromatic ring ttzbenz ligands (Koşar *et al.*, 2012 ▸), due to the close resemblance of the emission band of the two compounds. We also note downward absorption values ranging from compound (I) to (II), which may be due to the increase in the atomic number from Sr^2+^ to Ba^2+^.

## Refinement   

Crystal data, data collection and structure refinement details are summarized in Table 5 ▸. The water H atoms were located in a difference-Fourier map and their positions and isotropic displacement parameters were refined. All other H atoms were placed in geometrically idealized positions and constrained to ride on their parent atoms (C—H = 0.93 Å) with *U*_iso_(H) = 1.2*U*_eq_(C).

## Supplementary Material

Crystal structure: contains datablock(s) TTZBENZ_AE, I, II. DOI: 10.1107/S2056989020006386/tx2021sup1.cif

Structure factors: contains datablock(s) I. DOI: 10.1107/S2056989020006386/tx2021Isup2.hkl

Structure factors: contains datablock(s) II. DOI: 10.1107/S2056989020006386/tx2021IIsup3.hkl

Infrared spectra. DOI: 10.1107/S2056989020006386/tx2021sup4.jpg

Photoluminescent spectra. DOI: 10.1107/S2056989020006386/tx2021sup5.jpg

Table S1. Comparative shape analysis of two polymers. DOI: 10.1107/S2056989020006386/tx2021sup6.doc

CCDC references: 2003538, 2003537

Additional supporting information:  crystallographic information; 3D view; checkCIF report

## Figures and Tables

**Table 1:** Selected geometric parameters (Å, °) for (I)

Ba1—O1	2.6598 (17)	Ba1—O2	2.8750 (12)
Ba1—O1^i^	2.6598 (17)	Ba1—O2^iii^	2.8750 (12)
Ba1—O3^i^	2.821 (2)	Ba1—O2^i^	2.8750 (12)
Ba1—O3	2.821 (2)	Ba1—O1^ii^	3.0157 (17)
Ba1—O2^ii^	2.8750 (12)	Ba1—O1^iii^	3.0157 (17)
			
O1—Ba1—O1^i^	137.57 (7)	O2^ii^—Ba1—O2^iii^	142.501 (17)
O1—Ba1—O3^i^	75.09 (3)	O2—Ba1—O2^iii^	91.23 (5)
O3^i^—Ba1—O3	89.36 (11)	O1—Ba1—O1^ii^	132.46 (5)
O1—Ba1—O2^ii^	134.06 (2)	O3^i^—Ba1—O1^ii^	131.51 (5)
O1^i^—Ba1—O2^ii^	62.43 (2)	O2^ii^—Ba1—O1^ii^	58.35 (2)
O3^i^—Ba1—O2^ii^	74.10 (4)	O2—Ba1—O1^ii^	85.73 (2)
O3—Ba1—O2^ii^	136.98 (2)	O1^ii^—Ba1—O1^iii^	42.49 (6)
O2^ii^—Ba1—O2	76.81 (4)		

Symmetry codes: (i) 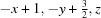; (ii) 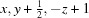; (iii) 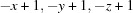.

**Table 2:** Selected geometric parameters (Å, °) for (II)

Sr—O1	2.501 (2)	Sr—O1^i^	2.6602 (14)
Sr—O3	2.522 (2)	Sr—O4	2.6757 (18)
Sr—O2	2.549 (3)		
			
O1—Sr—O1^ii^	140.67 (7)	O3—Sr—O1^iii^	148.91 (4)
O1—Sr—O3	85.19 (4)	O1—Sr—O4	68.20 (5)
O1—Sr—O2	72.67 (4)	O1^ii^—Sr—O4	147.71 (5)
O3—Sr—O2	103.31 (9)	O3—Sr—O4	83.72 (5)
O1—Sr—O1^i^	124.21 (4)	O2—Sr—O4	139.50 (4)
O1^ii^—Sr—O1^i^	77.42 (5)	O1^i^—Sr—O4	97.37 (4)
O3—Sr—O1^i^	148.91 (4)	O1^iii^—Sr—O4	66.00 (5)
O2—Sr—O1^i^	95.91 (7)	O1^iii^—Sr—O4^i^	97.37 (4)
O1^ii^—Sr—O1^iii^	124.21 (5)	O4—Sr—O4^i^	80.48 (7)

Symmetry codes: (i) 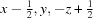; (ii) 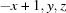; (iii) 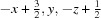.

**Table 3:** Hydrogen-bond geometry (Å, °) for (I)

*D*—H⋯*A*	*D*—H	H⋯*A*	*D*⋯*A*	*D*—H⋯*A*
O2—H2⋯N2^iv^	0.79 (2)	2.14 (2)	2.927 (2)	175 (3)
O3—H3⋯N1^v^	0.79 (3)	2.29 (3)	3.069 (2)	169 (3)

Symmetry codes: (iv) 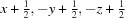; (v) 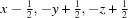.

**Table 4:** Hydrogen-bond geometry (Å, °) for (II)

*D*—H⋯*A*	*D*—H	H⋯*A*	*D*⋯*A*	*D*—H⋯*A*
O3—H3⋯N1^iv^	0.85 (2)	1.96 (2)	2.800 (2)	171 (3)
O3—H3⋯N2^iv^	0.85 (2)	2.62 (2)	3.314 (3)	141 (2)
O2—H2⋯N2^v^	0.77 (3)	2.53 (3)	3.270 (3)	160 (3)
O4—H4⋯N2^vi^	0.87 (2)	1.93 (2)	2.784 (2)	166 (2)

Symmetry codes: (iv) 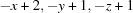; (v) 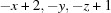; (vi) 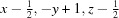.

**Table 5:** Experimental details

	(I)	(II)
Crystal data		
Chemical formula	[Ba(C_8_H_4_N_4_O_2_)(H_2_O)_4_]	[Sr(C_8_H_4_N_4_O_2_)(H_2_O)_3_]
*M*_r_	397.55	329.82
Crystal system, space group	Orthorhombic, *I* *m* *m* *a*	Orthorhombic, *P* *m* *n* *a*
Temperature (K)	298	150
*a*, *b*, *c* (Å)	7.5012 (1), 7.1444 (1), 24.7457 (5)	6.914 (6), 7.018 (7), 24.164 (2)
*V* (Å^3^)	1326.16 (4)	1172.5 (16)
*Z*	4	4
Radiation type	Mo *K*α	Mo *K*α
μ (mm^−1^)	3.02	4.62
Crystal size (mm)	0.6 × 0.5 × 0.22	0.20 × 0.1 × 0.07

Data collection		
Diffractometer	Bruker APEXII CCD	Bruker APEXII CCD
Absorption correction	Multi-scan (*SADABS*; Bruker, 2011 ▸)	Multi-scan (*SADABS*; Bruker, 2011 ▸)
*T*_min_, *T*_max_	0.670, 0.747	0.67, 0.747
No. of measured, independent and observed [*I* > 2σ(*I*)] reflections	5216, 952, 920	9495, 2091, 1740
*R*_int_	0.032	0.038
(sin θ/λ)_max_ (Å^−1^)	0.667	0.735

Refinement		
*R*[*F* ^2^ > 2σ(*F* ^2^)], *wR*(*F* ^2^), *S*	0.016, 0.039, 1.07	0.028, 0.062, 1.07
No. of reflections	937	2091
No. of parameters	70	105
No. of restraints	0	1
H-atom treatment	H atoms treated by a mixture of independent and constrained refinement	H atoms treated by a mixture of independent and constrained refinement
Δρ_max_, Δρ_min_ (e Å^−3^)	0.91, −0.31	0.65, −0.44

Computer programs: *APEX2* and *SAINT* (Bruker, 2011 ▸), *CrysAlis PRO* (Rigaku OD, 2015 ▸), *SHELXT* (Sheldrick, 2015*a* ▸), *SIR92* (Altomare *et al.*, 1993 ▸), *SHELXL* (Sheldrick, 2015*b* ▸), *SHELXL97* (Sheldrick, 2008 ▸), *ORTEP-3 for Windows* (Farrugia, 2012 ▸), *OLEX2* (Dolomanov *et al.*, 2009 ▸), *Mercury* (Macrae *et al.*, 2020 ▸), *PLATON* (Spek, 2020 ▸) and *publCIF* (Westrip, 2010 ▸).

## supplementary crystallographic information

### Poly[di-µ-aqua-diaqua[µ3-5-(4-carboxylatophenyl)-1H-1,2,3,4-tetrazol-1-ido-κ4O:O,O':O']barium(II)] (I) Crystal data

**Table:**

[Ba(C_8_H_4_N_4_O_2_)(H_2_O)_4_]	*F*(000) = 768
*M**_r_* = 397.55	*D*_x_ = 1.991 Mg m^−^^3^
Orthorhombic, *I**m**m**a*	Mo *K*α radiation, λ = 0.71073 Å
Hall symbol: -I 2b 2	Cell parameters from 9092 reflections
*a* = 7.5012 (1) Å	θ = 4.3–51.0°
*b* = 7.1444 (1) Å	µ = 3.02 mm^−^^1^
*c* = 24.7457 (5) Å	*T* = 298 K
*V* = 1326.16 (4) Å^3^	Block, colorless
*Z* = 4	0.6 × 0.5 × 0.22 mm

### Poly[di-µ-aqua-diaqua[µ3-5-(4-carboxylatophenyl)-1H-1,2,3,4-tetrazol-1-ido-κ4O:O,O':O']barium(II)] (I) Data collection

**Table:**

Bruker APEXII CCD diffractometer	920 reflections with *I* > 2σ(*I*)
φ and ω scans	*R*_int_ = 0.032
Absorption correction: multi-scan (SADABS; Bruker, 2011)	θ_max_ = 28.3°, θ_min_ = 4.9°
*T*_min_ = 0.670, *T*_max_ = 0.747	*h* = −10→7
5216 measured reflections	*k* = −9→7
952 independent reflections	*l* = −30→32

### Poly[di-µ-aqua-diaqua[µ3-5-(4-carboxylatophenyl)-1H-1,2,3,4-tetrazol-1-ido-κ4O:O,O':O']barium(II)] (I) Refinement

**Table:**

Refinement on *F*^2^	Primary atom site location: structure-invariant direct methods
Least-squares matrix: full	Secondary atom site location: difference Fourier map
*R*[*F*^2^ > 2σ(*F*^2^)] = 0.016	Hydrogen site location: mixed
*wR*(*F*^2^) = 0.039	H atoms treated by a mixture of independent and constrained refinement
*S* = 1.07	*w* = 1/[σ^2^(*F*_o_^2^) + (0.0155*P*)^2^ + 1.5691*P*] where *P* = (*F*_o_^2^ + 2*F*_c_^2^)/3
937 reflections	(Δ/σ)_max_ = 0.002
70 parameters	Δρ_max_ = 0.91 e Å^−^^3^
0 restraints	Δρ_min_ = −0.31 e Å^−^^3^

### Poly[di-µ-aqua-diaqua[µ3-5-(4-carboxylatophenyl)-1H-1,2,3,4-tetrazol-1-ido-κ4O:O,O':O']barium(II)] (I) Special details

**Table:**

Geometry. All esds (except the esd in the dihedral angle between two l.s. planes) are estimated using the full covariance matrix. The cell esds are taken into account individually in the estimation of esds in distances, angles and torsion angles; correlations between esds in cell parameters are only used when they are defined by crystal symmetry. An approximate (isotropic) treatment of cell esds is used for estimating esds involving l.s. planes.

### Poly[di-µ-aqua-diaqua[µ3-5-(4-carboxylatophenyl)-1H-1,2,3,4-tetrazol-1-ido-κ4O:O,O':O']barium(II)] (I) Fractional atomic coordinates and isotropic or equivalent isotropic displacement parameters (Å^2^)

**Table:**

	*x*	*y*	*z*	*U*_iso_*/*U*_eq_	
Ba1	0.5000	0.7500	0.462655 (6)	0.02050 (7)	
O2	0.7739 (2)	0.5000	0.5000	0.0309 (3)	
O1	0.5000	0.4029 (2)	0.42376 (7)	0.0385 (4)	
O3	0.2356 (3)	0.7500	0.38160 (10)	0.0458 (5)	
N2	0.5000	0.1584 (3)	0.08485 (7)	0.0312 (4)	
C1	0.5000	0.2500	0.39934 (11)	0.0197 (5)	
N1	0.5000	0.0959 (3)	0.13588 (7)	0.0313 (4)	
C2	0.5000	0.2500	0.33851 (11)	0.0221 (5)	
C3	0.5000	0.0832 (3)	0.31016 (9)	0.0343 (5)	
H3A	0.5000	−0.0298	0.3288	0.041*	
C5	0.5000	0.2500	0.22580 (12)	0.0243 (6)	
C6	0.5000	0.2500	0.16639 (12)	0.0233 (5)	
C4	0.5000	0.0829 (3)	0.25423 (9)	0.0369 (6)	
H4	0.5000	−0.0302	0.2356	0.044*	
H3	0.175 (4)	0.665 (4)	0.3725 (13)	0.072 (9)*	
H2	0.834 (3)	0.464 (4)	0.4761 (9)	0.046 (7)*	

### Poly[di-µ-aqua-diaqua[µ3-5-(4-carboxylatophenyl)-1H-1,2,3,4-tetrazol-1-ido-κ4O:O,O':O']barium(II)] (I) Atomic displacement parameters (Å^2^)

**Table:**

	*U*^11^	*U*^22^	*U*^33^	*U*^12^	*U*^13^	*U*^23^
Ba1	0.02865 (11)	0.01345 (10)	0.01940 (11)	0.000	0.000	0.000
O2	0.0294 (7)	0.0369 (9)	0.0265 (8)	0.000	0.000	−0.0053 (7)
O1	0.0744 (12)	0.0226 (8)	0.0184 (7)	0.000	0.000	−0.0041 (6)
O3	0.0508 (11)	0.0293 (9)	0.0571 (13)	0.000	−0.0202 (10)	0.000
N2	0.0469 (11)	0.0283 (10)	0.0183 (8)	0.000	0.000	−0.0020 (7)
C1	0.0246 (12)	0.0172 (12)	0.0173 (13)	0.000	0.000	0.000
N1	0.0527 (11)	0.0240 (9)	0.0171 (8)	0.000	0.000	−0.0010 (7)
C2	0.0319 (14)	0.0213 (13)	0.0132 (12)	0.000	0.000	0.000
C3	0.0659 (15)	0.0190 (9)	0.0180 (10)	0.000	0.000	0.0022 (8)
C5	0.0337 (14)	0.0231 (14)	0.0162 (13)	0.000	0.000	0.000
C6	0.0298 (13)	0.0223 (13)	0.0179 (13)	0.000	0.000	0.000
C4	0.0718 (17)	0.0193 (9)	0.0196 (10)	0.000	0.000	−0.0033 (8)

### Poly[di-µ-aqua-diaqua[µ3-5-(4-carboxylatophenyl)-1H-1,2,3,4-tetrazol-1-ido-κ4O:O,O':O']barium(II)] (I) Geometric parameters (Å, º)

**Table:**

Ba1—O1	2.6598 (17)	N2—N1	1.339 (3)
Ba1—O1^i^	2.6598 (17)	C1—O1^iv^	1.249 (2)
Ba1—O3^i^	2.821 (2)	C1—C2	1.505 (4)
Ba1—O3	2.821 (2)	N1—C6	1.335 (2)
Ba1—O2^ii^	2.8750 (12)	C2—C3^iv^	1.383 (3)
Ba1—O2	2.8750 (12)	C2—C3	1.383 (3)
Ba1—O2^iii^	2.8750 (12)	C3—C4	1.384 (3)
Ba1—O2^i^	2.8750 (12)	C3—H3A	0.9300
Ba1—O1^ii^	3.0157 (17)	C5—C4	1.386 (3)
Ba1—O1^iii^	3.0157 (17)	C5—C4^iv^	1.386 (3)
O2—H2	0.78 (2)	C5—C6	1.470 (4)
O1—C1	1.249 (2)	C6—N1^iv^	1.335 (2)
O3—H3	0.80 (3)	C4—H4	0.9300
N2—N2^iv^	1.308 (4)		
			
O1—Ba1—O1^i^	137.57 (7)	Ba1^iii^—O2—Ba1	88.77 (5)
O1—Ba1—O3^i^	75.09 (3)	Ba1^v^—O2—H2	117 (2)
O1^i^—Ba1—O3^i^	75.09 (3)	Ba1^vi^—O2—H2	117 (2)
O1—Ba1—O3	75.09 (3)	Ba1^vii^—O2—H2	117 (2)
O1^i^—Ba1—O3	75.09 (3)	Ba1^iii^—O2—H2	117 (2)
O3^i^—Ba1—O3	89.36 (11)	Ba1—O2—H2	111.5 (19)
O1—Ba1—O2^ii^	134.06 (2)	C1—O1—Ba1	172.27 (16)
O1^i^—Ba1—O2^ii^	62.43 (2)	C1—O1—Ba1^v^	97.70 (14)
O3^i^—Ba1—O2^ii^	74.10 (4)	Ba1—O1—Ba1^v^	90.03 (5)
O3—Ba1—O2^ii^	136.98 (2)	C1—O1—Ba1^vii^	97.70 (14)
O1—Ba1—O2	62.43 (2)	Ba1—O1—Ba1^vii^	90.03 (5)
O1^i^—Ba1—O2	134.06 (2)	C1—O1—Ba1^iii^	97.70 (14)
O3^i^—Ba1—O2	74.10 (4)	Ba1—O1—Ba1^iii^	90.03 (5)
O3—Ba1—O2	136.98 (2)	C1—O1—Ba1^vi^	97.70 (14)
O2^ii^—Ba1—O2	76.81 (4)	Ba1—O1—Ba1^vi^	90.03 (5)
O1—Ba1—O2^iii^	62.43 (2)	Ba1—O3—H3	127 (2)
O1^i^—Ba1—O2^iii^	134.06 (2)	N2^iv^—N2—N1	109.49 (12)
O3^i^—Ba1—O2^iii^	136.98 (2)	O1—C1—O1^iv^	122.1 (3)
O3—Ba1—O2^iii^	74.10 (4)	O1—C1—C2	118.95 (13)
O2^ii^—Ba1—O2^iii^	142.501 (17)	O1^iv^—C1—C2	118.95 (13)
O2—Ba1—O2^iii^	91.23 (5)	O1—C1—Ba1^vii^	61.05 (13)
O1—Ba1—O2^i^	134.06 (2)	O1^iv^—C1—Ba1^vii^	61.05 (13)
O1^i^—Ba1—O2^i^	62.43 (2)	C2—C1—Ba1^vii^	180.0
O3^i^—Ba1—O2^i^	136.98 (2)	O1—C1—Ba1^iii^	61.05 (13)
O3—Ba1—O2^i^	74.10 (4)	O1^iv^—C1—Ba1^iii^	61.05 (13)
O2^ii^—Ba1—O2^i^	91.23 (5)	C2—C1—Ba1^iii^	180.0
O2—Ba1—O2^i^	142.501 (17)	O1—C1—Ba1^vi^	61.05 (13)
O2^iii^—Ba1—O2^i^	76.81 (4)	O1^iv^—C1—Ba1^vi^	61.05 (13)
O1—Ba1—O1^ii^	132.46 (5)	C2—C1—Ba1^vi^	180.0
O1^i^—Ba1—O1^ii^	89.97 (5)	O1—C1—Ba1^v^	61.05 (13)
O3^i^—Ba1—O1^ii^	131.51 (5)	O1^iv^—C1—Ba1^v^	61.05 (13)
O3—Ba1—O1^ii^	131.51 (5)	C2—C1—Ba1^v^	180.0
O2^ii^—Ba1—O1^ii^	58.35 (2)	C6—N1—N2	104.94 (19)
O2—Ba1—O1^ii^	85.73 (2)	C3^iv^—C2—C3	119.0 (3)
O2^iii^—Ba1—O1^ii^	85.73 (2)	C3^iv^—C2—C1	120.48 (13)
O2^i^—Ba1—O1^ii^	58.35 (2)	C3—C2—C1	120.48 (13)
O1—Ba1—O1^iii^	89.97 (5)	C2—C3—C4	120.6 (2)
O1^i^—Ba1—O1^iii^	132.46 (5)	C2—C3—H3A	119.7
O3^i^—Ba1—O1^iii^	131.51 (5)	C4—C3—H3A	119.7
O3—Ba1—O1^iii^	131.51 (5)	C4—C5—C4^iv^	119.0 (3)
O2^ii^—Ba1—O1^iii^	85.73 (2)	C4—C5—C6	120.50 (14)
O2—Ba1—O1^iii^	58.35 (2)	C4^iv^—C5—C6	120.50 (14)
O2^iii^—Ba1—O1^iii^	58.35 (2)	N1^iv^—C6—N1	111.1 (3)
O2^i^—Ba1—O1^iii^	85.73 (2)	N1^iv^—C6—C5	124.44 (13)
O1^ii^—Ba1—O1^iii^	42.49 (6)	N1—C6—C5	124.44 (13)
Ba1^v^—O2—Ba1	88.77 (5)	C3—C4—C5	120.4 (2)
Ba1^vi^—O2—Ba1	88.77 (5)	C3—C4—H4	119.8
Ba1^vii^—O2—Ba1	88.77 (5)	C5—C4—H4	119.8
			
O1—Ba1—O2—Ba1^v^	−57.76 (3)	O1^ii^—Ba1—O2—Ba1^vii^	85.62 (2)
O1^i^—Ba1—O2—Ba1^v^	171.44 (5)	O1^iii^—Ba1—O2—Ba1^vii^	50.97 (3)
O3^i^—Ba1—O2—Ba1^v^	−138.96 (4)	O1—Ba1—O2—Ba1^iii^	−57.76 (3)
O3—Ba1—O2—Ba1^v^	−67.75 (7)	O1^i^—Ba1—O2—Ba1^iii^	171.44 (5)
O2^ii^—Ba1—O2—Ba1^v^	144.096 (12)	O3^i^—Ba1—O2—Ba1^iii^	−138.96 (4)
O2^i^—Ba1—O2—Ba1^v^	69.71 (4)	O3—Ba1—O2—Ba1^iii^	−67.75 (7)
O1^ii^—Ba1—O2—Ba1^v^	85.62 (2)	O2^ii^—Ba1—O2—Ba1^iii^	144.096 (12)
O1^iii^—Ba1—O2—Ba1^v^	50.97 (3)	O2^i^—Ba1—O2—Ba1^iii^	69.71 (4)
O1—Ba1—O2—Ba1^vi^	−57.76 (3)	O1^ii^—Ba1—O2—Ba1^iii^	85.62 (2)
O1^i^—Ba1—O2—Ba1^vi^	171.44 (5)	O1^iii^—Ba1—O2—Ba1^iii^	50.97 (3)
O3^i^—Ba1—O2—Ba1^vi^	−138.96 (4)	O3^i^—Ba1—O1—Ba1^v^	133.31 (5)
O3—Ba1—O2—Ba1^vi^	−67.75 (7)	O2^ii^—Ba1—O1—Ba1^v^	84.02 (4)
O2^ii^—Ba1—O2—Ba1^vi^	144.096 (12)	O2—Ba1—O1—Ba1^v^	53.73 (3)
O2^i^—Ba1—O2—Ba1^vi^	69.71 (4)	O3^i^—Ba1—O1—Ba1^vii^	133.31 (5)
O1^ii^—Ba1—O2—Ba1^vi^	85.62 (2)	O2^ii^—Ba1—O1—Ba1^vii^	84.02 (4)
O1^iii^—Ba1—O2—Ba1^vi^	50.97 (3)	O2—Ba1—O1—Ba1^vii^	53.73 (3)
O1—Ba1—O2—Ba1^vii^	−57.76 (3)	O3^i^—Ba1—O1—Ba1^iii^	133.31 (5)
O1^i^—Ba1—O2—Ba1^vii^	171.44 (5)	O2^ii^—Ba1—O1—Ba1^iii^	84.02 (4)
O3^i^—Ba1—O2—Ba1^vii^	−138.96 (4)	O2—Ba1—O1—Ba1^iii^	53.73 (3)
O3—Ba1—O2—Ba1^vii^	−67.75 (7)	O3^i^—Ba1—O1—Ba1^vi^	133.31 (5)
O2^ii^—Ba1—O2—Ba1^vii^	144.096 (12)	O2^ii^—Ba1—O1—Ba1^vi^	84.02 (4)
O2^i^—Ba1—O2—Ba1^vii^	69.71 (4)	O2—Ba1—O1—Ba1^vi^	53.73 (3)

Symmetry codes: (i) −*x*+1, −*y*+3/2, *z*; (ii) *x*, *y*+1/2, −*z*+1; (iii) −*x*+1, −*y*+1, −*z*+1; (iv) −*x*+1, −*y*+1/2, *z*; (v) −*x*+1, *y*−1/2, −*z*+1; (vi) *x*, *y*−1/2, −*z*+1; (vii) *x*, −*y*+1, −*z*+1.

### Poly[di-µ-aqua-diaqua[µ3-5-(4-carboxylatophenyl)-1H-1,2,3,4-tetrazol-1-ido-κ4O:O,O':O']barium(II)] (I) Hydrogen-bond geometry (Å, º)

**Table:**

*D*—H···*A*	*D*—H	H···*A*	*D*···*A*	*D*—H···*A*
O2—H2···N2^viii^	0.79 (2)	2.14 (2)	2.927 (2)	175 (3)
O3—H3···N1^ix^	0.79 (3)	2.29 (3)	3.069 (2)	169 (3)

Symmetry codes: (viii) *x*+1/2, −*y*+1/2, −*z*+1/2; (ix) *x*−1/2, −*y*+1/2, −*z*+1/2.

### Poly[µ-aqua-diaqua[µ3-5-(4-carboxylatophenyl)-1H-1,2,3,4-tetrazol-1-ido-κ4O:O,O':O']strontium(II)] (II) Crystal data

**Table:**

[Sr(C_8_H_4_N_4_O_2_)(H_2_O)_3_]	*F*(000) = 656
*M**_r_* = 329.82	*D*_x_ = 1.874 Mg m^−^^3^
Orthorhombic, *P**m**n**a*	Mo *K*α radiation, λ = 0.71073 Å
Hall symbol: -P 2ac 2	Cell parameters from 10707 reflections
*a* = 6.914 (6) Å	θ = 4.9–34.3°
*b* = 7.018 (7) Å	µ = 4.62 mm^−^^1^
*c* = 24.164 (2) Å	*T* = 150 K
*V* = 1172.5 (16) Å^3^	Prism, colorless
*Z* = 4	0.20 × 0.1 × 0.07 mm

### Poly[µ-aqua-diaqua[µ3-5-(4-carboxylatophenyl)-1H-1,2,3,4-tetrazol-1-ido-κ4O:O,O':O']strontium(II)] (II) Data collection

**Table:**

Bruker APEXII CCD diffractometer	2091 independent reflections
Radiation source: fine-focus sealed tube	1740 reflections with *I* > 2σ(*I*)
Graphite monochromator	*R*_int_ = 0.038
φ and ω scans	θ_max_ = 31.5°, θ_min_ = 3.4°
Absorption correction: multi-scan (SADABS; Bruker, 2011)	*h* = −10→8
*T*_min_ = 0.67, *T*_max_ = 0.747	*k* = −10→8
9495 measured reflections	*l* = −34→35

### Poly[µ-aqua-diaqua[µ3-5-(4-carboxylatophenyl)-1H-1,2,3,4-tetrazol-1-ido-κ4O:O,O':O']strontium(II)] (II) Refinement

**Table:**

Refinement on *F*^2^	1 restraint
Least-squares matrix: full	H atoms treated by a mixture of independent and constrained refinement
*R*[*F*^2^ > 2σ(*F*^2^)] = 0.028	*w* = 1/[σ^2^(*F*_o_^2^) + (0.0253*P*)^2^ + 0.7105*P*] where *P* = (*F*_o_^2^ + 2*F*_c_^2^)/3
*wR*(*F*^2^) = 0.062	(Δ/σ)_max_ = 0.001
*S* = 1.07	Δρ_max_ = 0.65 e Å^−^^3^
2091 reflections	Δρ_min_ = −0.44 e Å^−^^3^
105 parameters	

### Poly[µ-aqua-diaqua[µ3-5-(4-carboxylatophenyl)-1H-1,2,3,4-tetrazol-1-ido-κ4O:O,O':O']strontium(II)] (II) Special details

**Table:**

Geometry. All esds (except the esd in the dihedral angle between two l.s. planes) are estimated using the full covariance matrix. The cell esds are taken into account individually in the estimation of esds in distances, angles and torsion angles; correlations between esds in cell parameters are only used when they are defined by crystal symmetry. An approximate (isotropic) treatment of cell esds is used for estimating esds involving l.s. planes.

### Poly[µ-aqua-diaqua[µ3-5-(4-carboxylatophenyl)-1H-1,2,3,4-tetrazol-1-ido-κ4O:O,O':O']strontium(II)] (II) Fractional atomic coordinates and isotropic or equivalent isotropic displacement parameters (Å^2^)

**Table:**

	*x*	*y*	*z*	*U*_iso_*/*U*_eq_	
Sr	0.5000	0.41161 (3)	0.285826 (9)	0.01071 (7)	
O4	0.7500	0.6752 (3)	0.2500	0.0148 (4)	
O2	0.5000	0.0703 (3)	0.32193 (13)	0.0341 (6)	
O3	0.5000	0.6090 (3)	0.37304 (8)	0.0201 (4)	
O1	0.84065 (19)	0.3310 (2)	0.31160 (5)	0.0157 (3)	
C2	1.0000	0.2947 (3)	0.39842 (10)	0.0112 (5)	
N1	1.1596 (2)	0.1898 (2)	0.60389 (6)	0.0155 (3)	
C4	0.8268 (3)	0.2547 (3)	0.48414 (7)	0.0166 (4)	
H4A	0.7101	0.2459	0.5031	0.020*	
C5	1.0000	0.2407 (4)	0.51296 (10)	0.0120 (5)	
N2	1.0952 (2)	0.1577 (2)	0.65534 (6)	0.0167 (3)	
C6	1.0000	0.2079 (3)	0.57327 (10)	0.0120 (5)	
C3	0.8269 (3)	0.2818 (3)	0.42714 (7)	0.0166 (4)	
H3A	0.7102	0.2912	0.4082	0.020*	
C1	1.0000	0.3206 (4)	0.33702 (10)	0.0113 (5)	
H3	0.600 (3)	0.668 (4)	0.3839 (10)	0.037 (7)*	
H4	0.709 (4)	0.743 (4)	0.2212 (9)	0.036 (8)*	
H2	0.584 (4)	0.014 (5)	0.3353 (12)	0.058 (10)*	

### Poly[µ-aqua-diaqua[µ3-5-(4-carboxylatophenyl)-1H-1,2,3,4-tetrazol-1-ido-κ4O:O,O':O']strontium(II)] (II) Atomic displacement parameters (Å^2^)

**Table:**

	*U*^11^	*U*^22^	*U*^33^	*U*^12^	*U*^13^	*U*^23^
Sr	0.00658 (11)	0.01630 (11)	0.00923 (10)	0.000	0.000	0.00006 (10)
O4	0.0141 (10)	0.0173 (8)	0.0131 (9)	0.000	−0.0015 (7)	0.000
O2	0.0211 (13)	0.0223 (12)	0.0590 (18)	0.000	0.000	0.0144 (12)
O3	0.0102 (10)	0.0302 (11)	0.0200 (10)	0.000	0.000	−0.0087 (9)
O1	0.0088 (6)	0.0258 (7)	0.0124 (6)	0.0014 (6)	−0.0017 (5)	0.0045 (5)
C2	0.0120 (12)	0.0107 (10)	0.0107 (11)	0.000	0.000	0.0017 (9)
N1	0.0144 (8)	0.0215 (8)	0.0106 (7)	−0.0015 (6)	−0.0014 (6)	0.0002 (6)
C4	0.0101 (9)	0.0251 (9)	0.0146 (8)	0.0019 (8)	0.0022 (7)	0.0017 (7)
C5	0.0140 (12)	0.0126 (11)	0.0093 (11)	0.000	0.000	−0.0019 (9)
N2	0.0186 (8)	0.0206 (7)	0.0110 (7)	−0.0015 (7)	−0.0010 (6)	0.0000 (6)
C6	0.0134 (12)	0.0112 (10)	0.0114 (11)	0.000	0.000	−0.0019 (9)
C3	0.0102 (9)	0.0260 (9)	0.0137 (8)	0.0023 (7)	−0.0009 (7)	0.0026 (7)
C1	0.0084 (12)	0.0124 (11)	0.0132 (11)	0.000	0.000	0.0007 (9)

### Poly[µ-aqua-diaqua[µ3-5-(4-carboxylatophenyl)-1H-1,2,3,4-tetrazol-1-ido-κ4O:O,O':O']strontium(II)] (II) Geometric parameters (Å, º)

**Table:**

Sr—O1	2.501 (2)	C2—C3	1.387 (2)
Sr—O1^i^	2.501 (2)	C2—C1	1.495 (3)
Sr—O3	2.522 (2)	N1—C6	1.335 (2)
Sr—O2	2.549 (3)	N1—N2	1.340 (2)
Sr—O1^ii^	2.6602 (14)	C4—C5	1.389 (2)
Sr—O1^iii^	2.6602 (14)	C4—C3	1.390 (2)
Sr—O4	2.6757 (18)	C4—H4A	0.9300
Sr—O4^ii^	2.6757 (18)	C5—C4^iv^	1.389 (2)
O4—H4	0.89 (2)	C5—C6	1.475 (3)
O2—H2	0.77 (3)	N2—N2^iv^	1.316 (4)
O3—H3	0.846 (16)	C6—N1^iv^	1.335 (2)
O1—C1	1.2635 (19)	C3—H3A	0.9300
C2—C3^iv^	1.387 (2)	C1—O1^iv^	1.2635 (19)
			
O1—Sr—O1^i^	140.67 (7)	O4^ii^—Sr—Sr^ii^	43.74 (4)
O1—Sr—O3	85.19 (4)	C1^ii^—Sr—Sr^ii^	64.037 (19)
O1^i^—Sr—O3	85.20 (4)	O1—Sr—Sr^v^	43.07 (4)
O1—Sr—O2	72.67 (4)	O1^i^—Sr—Sr^v^	162.46 (3)
O1^i^—Sr—O2	72.67 (4)	O3—Sr—Sr^v^	111.97 (2)
O3—Sr—O2	103.31 (9)	O2—Sr—Sr^v^	98.82 (3)
O1—Sr—O1^ii^	124.21 (4)	O1^ii^—Sr—Sr^v^	88.51 (4)
O1^i^—Sr—O1^ii^	77.42 (5)	O1^iii^—Sr—Sr^v^	39.95 (3)
O3—Sr—O1^ii^	148.91 (4)	O4—Sr—Sr^v^	43.74 (4)
O2—Sr—O1^ii^	95.91 (7)	O4^ii^—Sr—Sr^v^	115.64 (3)
O1—Sr—O1^iii^	77.42 (5)	C1^ii^—Sr—Sr^v^	64.037 (19)
O1^i^—Sr—O1^iii^	124.21 (5)	Sr^ii^—Sr—Sr^v^	126.79 (4)
O3—Sr—O1^iii^	148.91 (4)	Sr—O4—Sr^v^	92.52 (8)
O2—Sr—O1^iii^	95.91 (7)	Sr—O4—H4	114.6 (18)
O1^ii^—Sr—O1^iii^	48.93 (6)	Sr^v^—O4—H4	108.9 (17)
O1—Sr—O4	68.20 (5)	Sr—O2—H2	129 (2)
O1^i^—Sr—O4	147.71 (5)	Sr—O3—H3	121.9 (18)
O3—Sr—O4	83.72 (5)	C1—O1—Sr	162.79 (14)
O2—Sr—O4	139.50 (4)	C1—O1—Sr^v^	94.66 (12)
O1^ii^—Sr—O4	97.37 (4)	Sr—O1—Sr^v^	96.98 (5)
O1^iii^—Sr—O4	66.00 (5)	C3^iv^—C2—C3	119.4 (2)
O1—Sr—O4^ii^	147.71 (5)	C3^iv^—C2—C1	120.32 (11)
O1^i^—Sr—O4^ii^	68.20 (5)	C3—C2—C1	120.32 (11)
O3—Sr—O4^ii^	83.72 (5)	C6—N1—N2	104.81 (16)
O2—Sr—O4^ii^	139.50 (4)	C5—C4—C3	120.41 (18)
O1^ii^—Sr—O4^ii^	66.00 (5)	C5—C4—H4A	119.8
O1^iii^—Sr—O4^ii^	97.37 (4)	C3—C4—H4A	119.8
O4—Sr—O4^ii^	80.48 (7)	C4—C5—C4^iv^	119.1 (2)
O1—Sr—C1^ii^	101.29 (3)	C4—C5—C6	120.42 (11)
O1^i^—Sr—C1^ii^	101.29 (3)	C4^iv^—C5—C6	120.42 (11)
O3—Sr—C1^ii^	158.83 (7)	N2^iv^—N2—N1	109.42 (10)
O2—Sr—C1^ii^	97.87 (9)	N1^iv^—C6—N1	111.5 (2)
O1^ii^—Sr—C1^ii^	24.50 (3)	N1^iv^—C6—C5	124.22 (11)
O1^iii^—Sr—C1^ii^	24.50 (3)	N1—C6—C5	124.22 (11)
O4—Sr—C1^ii^	80.16 (4)	C2—C3—C4	120.34 (18)
O4^ii^—Sr—C1^ii^	80.16 (4)	C2—C3—H3A	119.8
O1—Sr—Sr^ii^	162.46 (3)	C4—C3—H3A	119.8
O1^i^—Sr—Sr^ii^	43.07 (4)	O1—C1—O1^iv^	121.4 (2)
O3—Sr—Sr^ii^	111.97 (2)	O1—C1—C2	119.31 (11)
O2—Sr—Sr^ii^	98.82 (3)	O1^iv^—C1—C2	119.31 (11)
O1^ii^—Sr—Sr^ii^	39.95 (3)	O1—C1—Sr^v^	60.84 (11)
O1^iii^—Sr—Sr^ii^	88.51 (4)	O1^iv^—C1—Sr^v^	60.84 (11)
O4—Sr—Sr^ii^	115.64 (3)	C2—C1—Sr^v^	174.84 (17)
			
O1—Sr—O4—Sr^v^	43.94 (4)	O4^ii^—Sr—O1—Sr^v^	−59.57 (8)
O1^i^—Sr—O4—Sr^v^	−158.12 (6)	C1^ii^—Sr—O1—Sr^v^	29.86 (6)
O3—Sr—O4—Sr^v^	131.27 (4)	Sr^ii^—Sr—O1—Sr^v^	61.70 (12)
O2—Sr—O4—Sr^v^	28.11 (11)	C3—C4—C5—C4^iv^	0.2 (4)
O1^ii^—Sr—O4—Sr^v^	−80.03 (4)	C3—C4—C5—C6	−178.7 (2)
O1^iii^—Sr—O4—Sr^v^	−41.54 (3)	C6—N1—N2—N2^iv^	−0.35 (16)
O4^ii^—Sr—O4—Sr^v^	−144.08 (2)	N2—N1—C6—N1^iv^	0.6 (3)
C1^ii^—Sr—O4—Sr^v^	−62.52 (4)	N2—N1—C6—C5	−178.6 (2)
Sr^ii^—Sr—O4—Sr^v^	−117.34 (3)	C4—C5—C6—N1^iv^	−0.1 (4)
O1^i^—Sr—O1—C1	−74.0 (5)	C4^iv^—C5—C6—N1^iv^	−179.0 (2)
O3—Sr—O1—C1	2.4 (5)	C4—C5—C6—N1	179.0 (2)
O2—Sr—O1—C1	−103.1 (5)	C4^iv^—C5—C6—N1	0.1 (4)
O1^ii^—Sr—O1—C1	171.6 (5)	C3^iv^—C2—C3—C4	−0.5 (4)
O1^iii^—Sr—O1—C1	156.5 (5)	C1—C2—C3—C4	179.0 (2)
O4—Sr—O1—C1	87.6 (5)	C5—C4—C3—C2	0.1 (3)
O4^ii^—Sr—O1—C1	72.7 (5)	Sr—O1—C1—O1^iv^	−138.6 (3)
C1^ii^—Sr—O1—C1	162.1 (5)	Sr^v^—O1—C1—O1^iv^	−6.2 (3)
Sr^ii^—Sr—O1—C1	−166.1 (4)	Sr—O1—C1—C2	41.6 (6)
Sr^v^—Sr—O1—C1	132.2 (5)	Sr^v^—O1—C1—C2	174.1 (2)
O1^i^—Sr—O1—Sr^v^	153.83 (6)	Sr—O1—C1—Sr^v^	−132.5 (5)
O3—Sr—O1—Sr^v^	−129.77 (7)	C3^iv^—C2—C1—O1	179.6 (2)
O2—Sr—O1—Sr^v^	124.67 (8)	C3—C2—C1—O1	0.1 (4)
O1^ii^—Sr—O1—Sr^v^	39.36 (9)	C3^iv^—C2—C1—O1^iv^	−0.1 (4)
O1^iii^—Sr—O1—Sr^v^	24.29 (6)	C3—C2—C1—O1^iv^	−179.6 (2)
O4—Sr—O1—Sr^v^	−44.63 (4)		

Symmetry codes: (i) −*x*+1, *y*, *z*; (ii) *x*−1/2, *y*, −*z*+1/2; (iii) −*x*+3/2, *y*, −*z*+1/2; (iv) −*x*+2, *y*, *z*; (v) *x*+1/2, *y*, −*z*+1/2.

### Poly[µ-aqua-diaqua[µ3-5-(4-carboxylatophenyl)-1H-1,2,3,4-tetrazol-1-ido-κ4O:O,O':O']strontium(II)] (II) Hydrogen-bond geometry (Å, º)

**Table:**

*D*—H···*A*	*D*—H	H···*A*	*D*···*A*	*D*—H···*A*
O3—H3···N1^vi^	0.85 (2)	1.96 (2)	2.800 (2)	171 (3)
O3—H3···N2^vi^	0.85 (2)	2.62 (2)	3.314 (3)	141 (2)
O2—H2···N2^vii^	0.77 (3)	2.53 (3)	3.270 (3)	160 (3)
O4—H4···N2^viii^	0.87 (2)	1.93 (2)	2.784 (2)	166 (2)

Symmetry codes: (vi) −*x*+2, −*y*+1, −*z*+1; (vii) −*x*+2, −*y*, −*z*+1; (viii) *x*−1/2, −*y*+1, *z*−1/2.
