# Supplementary figures and images for: In situ synthesis, crystal structures, topology and photoluminescent properties of poly[di-μ-aqua-di­aqua­[μ3-4-(1H-tetra­zol-1-id-5-yl)benzoato-κ4 O:O,O′:O′′]barium(II)] and poly[μ-aqua-di­aqua­[μ3-4-(1H-tetra­zol-1-id-5-yl)benzoato-κ4 O:O,O′:O′]strontium(II)]
Source: Acta Crystallogr E Crystallogr Commun. 2020 May 19;76(Pt 6):877–83. doi: 10.1107/S2056989020006386 (PMC7273978; doi:10.1107/S2056989020006386)

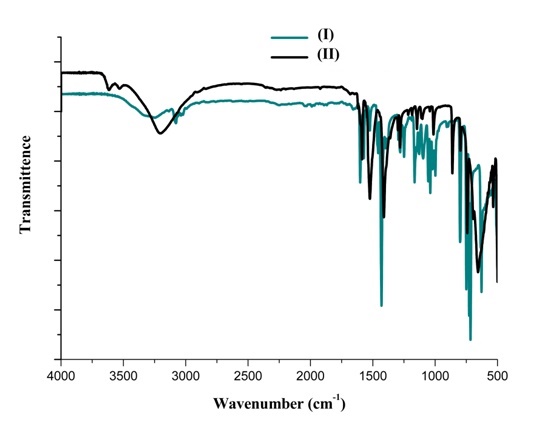

Supplement: Supplementary file 4 [file e-76-00877-sup4.jpg]

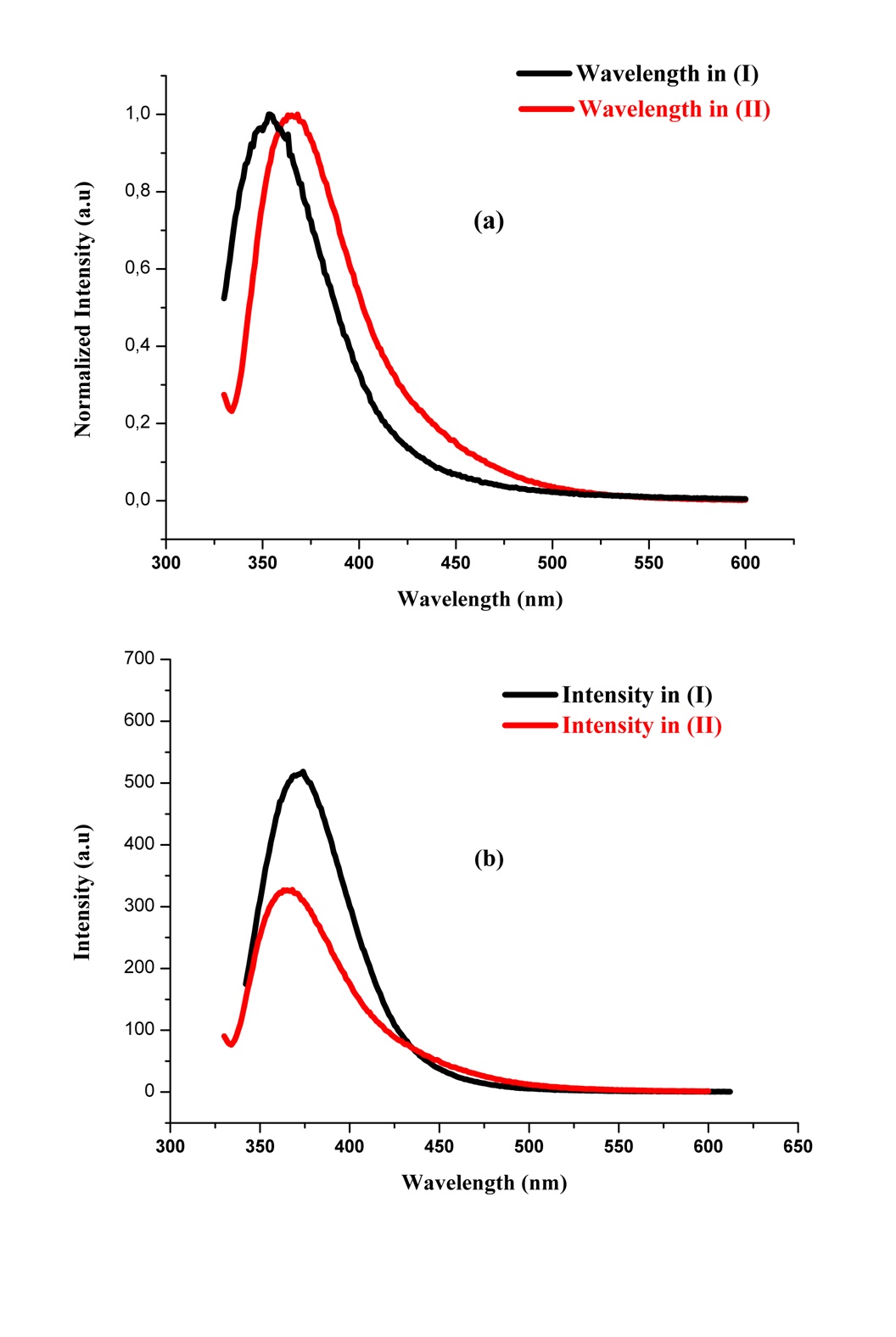

Supplement: Supplementary file 5 [file e-76-00877-sup5.jpg]
