# Supplementary material for: In situ synthesis, crystal structures, topology and photoluminescent properties of poly[di-μ-aqua-di­aqua­[μ3-4-(1H-tetra­zol-1-id-5-yl)benzoato-κ4 O:O,O′:O′′]barium(II)] and poly[μ-aqua-di­aqua­[μ3-4-(1H-tetra­zol-1-id-5-yl)benzoato-κ4 O:O,O′:O′]strontium(II)]
Source: Acta Crystallogr E Crystallogr Commun. 2020 May 19;76(Pt 6):877–83. doi: 10.1107/S2056989020006386 (PMC7273978; doi:10.1107/S2056989020006386)
Supplement: Supplementary file 6 [file e-76-00877-sup6.doc]

**Supplementary material table S-1**

**Comparative study of shape analysis between two polymers using SHAPE V2.1 software (Casanova, et al., 2005).**

*The minimum is taken for all possible relative orientations in space, and for all possible pairings of the vertices of the problem and reference polyhedra. Calculated from :*

*
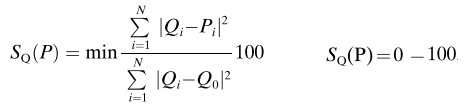
*

*Where Pi is the position vector of the corresponding vertex in the reference polyhedron P, and Q0 the position vector of the geometrical center of the problem structure.*

*Chem. Eur. J. 2005, 11, 1479–1494*

| **Structure [C.N=10]*^(**)^*** | **Polymer I** | **Structure [C.N=8]*^(**)^*** | **Polymer II** | **Polymer III** |
| --- | --- | --- | --- | --- |
| **DP-10** | 34.573 | **OP-8** | 32.627 | 33.303 |
| **EPY-10** | 26.195 | **HPY-8** | 21.531 | 21.053 |
| **OBPY-10** | 15.984 | **HBPY-8** | 16.966 | 17.161 |
| **PPR-10** | 11.188 | **CU-8** | 12.662 | 12.145 |
| **PAPR-10** | 15.899 | **SAPR-8** | 3.618 | 2.746 |
| **JBCCU-10** | 17.231 | **TDD-8*^(*)^*** | **3.426** | 3.028 |
| **JBCSAPR-10** | 7.844 | **JGBF-8** | 15.989 | 16.095 |
| **JMBIC-10** | 10.873 | **JETBPY-8** | 25.524 | 25.396 |
| **JATDI-10** | 17.864 | **JBTPR-8** | 4.529 | 3.960 |
| **JSPC-10*^(*)^*** | **4.424** | BTPR-8 | 3.989 | 3.483 |
| **SDD-10** | 9.278 | **JSD-8** | 6.327 | 5.823 |
| **TD-10** | 8.555 | **TT-8** | 13.173 | 12.721 |
| **HD-10** | 14.182 | **ETBPY-8** | 21.558 | 21.485 |
| ***^(*)^JSPC-10: Sphenocorona, TDD-8 : Triangular dodecahedron***  **(**)**   | **Ideal structures ML10**  **DP-10** D10h Decagon  **EPY-10** C9v Enneagonal pyramid  **OBPY-10** D8h Octagonal bipyramid  **PPR-10** D5h Pentagonal prism  **PAPR-10** D5d Pentagonal antiprism  **JBCCU-10** D4h Bicapped cube J15  **JBCSAPR-10** D4d Bicapped square antiprism J17  **JMBIC-10** C2v Metabidiminished icosahedron J62  **JATDI-10** C3v Augmented tridiminished icosahedron J64  **JSPC-10** C2v Sphenocorona J87  **SDD-10** D2 Staggered Dodecahedron (2:6:2)  **TD-10** C2v Tetradecahedron (2:6:2)  **HD-10** D4h Hexadecahedron (2:6:2) or (1:4:4:1)  **Ideal structures ML8**  **OP-8** 1 D8h Octagon  **HPY-8** 2 C7v Heptagonal pyramid  **HBPY-8** 3 D6h Hexagonal bipyramid  **CU-8** 4 Oh Cube  **SAPR-8** 5 D4d Square antiprism  **TDD-8** 6 D2d Triangular dodecahedron  **JGBF-8** 7 D2d Johnson gyrobifastigium J26  **JETBPY-8** 8 D3h Johnson elongated triangular bipyramid J14  **JBTPR-8** 9 C2v Biaugmented trigonal prism J50  **BTPR-8** 10 C2v Biaugmented trigonal prism  **JSD-8** 11 D2d Snub diphenoid J84  **TT-8** 12 Td Triakis tetrahedron  **ETBPY-8** 13 D3h Elongated trigonal bipyramid | | --- | | | | | |
